# Nonstructural Protein NS4 of *Rice Stripe Virus* Plays a Critical Role in Viral Spread in the Body of Vector Insects

**DOI:** 10.1371/journal.pone.0088636

**Published:** 2014-02-11

**Authors:** Wei Wu, Limin Zheng, Hongyan Chen, Dongsheng Jia, Fan Li, Taiyun Wei

**Affiliations:** 1 Fujian Province Key Laboratory of Plant Virology, Institute of Plant Virology, Fujian Agriculture and Forestry University, Fuzhou, Fujian, PR China; 2 Key Laboratory of Agricultural Biodiversity for Pest Management of China’s Ministry of Education, Yunnan Agricultural University, Kunming, Yunnan, PR China; University of Louisville, United States of America

## Abstract

*Rice stripe virus* (RSV), a tenuivirus, is transmitted by small brown planthopper (SBPH) in a persistent-propagative manner. In this study, sequential infection of RSV in the internal organs of SBPH after ingestion of virus indicated that RSV initially infected the midgut epithelium, and then progressed to the visceral muscle tissues, through which RSV spread to the entire alimentary canal. Finally, RSV spread into the salivary glands and reproductive system. During viral infection, the nonstructural protein NS4 of RSV formed cytoplasmic inclusions in various tissues of viruliferous SBPH. We demonstrated that the ribonucleoprotein particles of RSV were closely associated with NS4-specific inclusions in the body of viruliferous SBPH through a direct interaction between NS4 and nucleoprotein of RSV. Moreover, the knockdown of NS4 expression due to RNA interference induced by dsRNA from *NS4* gene significantly prevented the spread of RSV in the bodies of SBPHs without a significant effect on viral replication in continuous cell culture derived from SBPH. All these results suggest that the nonstructural protein NS4 of RSV plays a critical role in viral spread by the vector insects.

## Introduction

Most plant viruses are transmitted by vector insects, which have been classified as nonpersistent, semipersistent and persistent manners [1], [2]. In persistent transmission, some viruses could propagate in vector insects. The replication of persistent-propagative plant viruses in the body of vector insects induces the formation of various cytopathological inclusions composed of nonstructural viral proteins [1], [2]. However, the functional roles of these viral inclusions in viral propagation in vector insects are not well understood due to the lack of a reverse genetics system.

The type species of the genus *Tenuivirus*, *Rice stripe virus* (RSV), is transmitted by the small brown planthopper (SBPH), *Laodelphax striatellus* Fallén, in a persistent-propagative and transovarial manner [3], [4]. RSV has recently spread rapidly throughout China, Korea and Japan [5], [6]. RSV has filamentous ribonucleoprotein particles (RNPs) that contain viral RNAs, nucleoprotein and RNA-dependent RNA polymerase (RdRP) [3]. A single open reading frame on RNA1 encodes RdRP [7]. The other three RNAs use an ambisense coding strategy, i.e. both the viral-sense RNA (vRNA) and viral complementary-sense RNA (vcRNA) have coding capacity [6]. vRNA2 encodes NS2, a viral RNA-silencing suppressor, and vcRNA2 encodes NSvc2, whose function is unknown [8], [9]. RNA3 encodes NS3, a nonstructural protein involved in viral replication or assembly, and NSvc3, a nucleoprotein [10]–[12]. RNA4 encodes NS4, a nonstructural protein, and NSvc4, a viral movement protein [13]–[15]. However, the functional roles of these proteins during viral infection in the body of vector insect are still unknown.

Previous studies indicated that filamentous electron-opaque (FEO) or amorphous semi-electron-opaque (ASO) inclusion bodies, constructed by nonstructural protein NS4 (NS4-specific inclusions), abundantly accumulated in the cytoplasm of virus-infected plant cells, and were also distributed in the lumen and epithelium of midgut, salivary gland and ovarioles of viruliferous SBPHs [16], [17]. Furthermore, some of these inclusion bodies contained RNPs of RSV [16], raising the possibility that NS4-specific inclusions might serve as the sites for viral replication or spread in vector insects.

To further investigate the functional roles of NS4-specific inclusions during viral persistent infection in the vector insects of RSV, here, we used RNA interference (RNAi) induced by double-stranded RNAs (dsRNAs) for knockdown of the expression of NS4 in the body of SBPHs [18]. Previously, dsRNAs expressed as hairpin RNA constructs directed against viral genes could induce RNAi for interference with the expression of viral genes in rice plants [19]. Transgenic plants that harboured hairpin RNA construct for *NS4* gene, which is located at the 5’ end of vRNA4, was susceptible to infection by RSV [19]. By contrast, RNAi induced by hairpin RNA construct for *NSvc4* gene, which is located at the 5’ end of vcRNA4, resulted in resistance [19]. Thus, RNAi induced by dsRNAs against viral gene could silence viral mRNA in a sequence-specific way. These results suggested that NS4 of RSV might not be essential for viral proliferation in rice plants, but be necessary for viral proliferation in vector insects [19]. Our findings revealed that the ingestion of synthesized dsRNA targeting viral gene from the nonstructural proteins of Southern rice black-streaked dwarf virus, a fijivirus, and *Rice ragged stunt virus* (RRSV), an orzavirus, specifically prevented viral infection in the body of vector insects [20], [21]. Recently, the system of dsRNA-mediated gene silencing has been established in the bodies of SBPHs [22], providing an opportunity for us to investigate the functional role of NS4 in the infection cycle of RSV in its vector insect.

In the present study, by combining an immunofluorescence technique and a feeding-based RNAi technique, we determined that RSV has evolved to exploit NS4-specific inclusions to facilitate viral spread in the body of viruliferous SBPH vector.

## Materials and Methods

### Ethics statement

No specific permits were required for the described field studies. We confirm that the location for samples collection is not privately owned or protected in any way, and that the field studies did not involve endangered or protected species.

### Virus, insects and antibodies

RSV samples were collected in rice fields from Fujian Province in eastern China. The RSV isolate was maintained on rice plants via transmission by SBPHs, as described previously [23]. Polyclonal antibodies against the nonstructural protein NS4 of RSV and against the purified RNPs of RSV were prepared as described previously [24], [25]. IgG was isolated from polyclonal antibodies and then was directly conjugated to fluorescein isothiocyanate (FITC) or rhodamine (Invitrogen), as described previously [26].

### Immunofluorescence labelling of internal organs of SBPHs after viral ingestion

Second instar nymphs of SBPHs were kept on diseased rice plants for 2 days. At different days after viral ingestion by SBPHs, internal organs of SBPHs were dissected and fixed with 4% paraformaldehyde for 2 h. After fixation, internal organs were performed for immunofluorescence microscopy, as described previously [26], [27]. RNPs of RSV were labelled with RNP-specific IgG directly conjugated to rhodamine (RNP-rhodamine); NS4-specific inclusions were labelled with NS4-specific IgG directly conjugated to FITC (NS4-FITC); actin was labelled with the actin dye phalloidin-FITC or phalloidin-Alexa Fluor 647 carboxylic acid (Invitrogen). The samples were examined by confocal microscopy, as described previously [26], [27].

### Yeast two-hybrid assay

Yeast two-hybrid assay was performed according to the instructions of the DUALmembrane starter kit user manual (Dualsystems Biotech.). Briefly, the combination of the bait vector pBT3-STE-NS4 and the prey vector pPR3-N-NSvc3 were co-transformed into yeast strain NMY51. The vector pTSU2-APP was co-transformed with the vector pNubG-Fe65 or the vector pPR3-N as positive or negative controls, respectively. All transformants were grown on SD-trp-leu-his-ade agar plates or performed by the HTX-galactosidase assay according to the instructions of the DUALmembrane starter kit user manual.

### dsRNA synthesis

The PCR products of a 543-bp segment of *NS4* gene of RSV and a 717-bp segment of green fluorescence protein (GFP) gene were amplified by PCR. The PCR products were used to synthesize dsRNAs *in vitro* according to the protocol of the T7 RiboMAX™ Express RNAi System kit (Promega).

### Examination of the effect of dsRNAs on viral accumulation and transmission by SBPHs

The membrane feeding approach to deliver dsRNAs targeting viral genes to SBPHs was performed as described previously [20], [21], [28]. Briefly, second instar nymphs of SBPHs were maintained with the mixed diet containing 0.5 µg/μl dsRNAs for 1 day via membrane feeding, fed on diseased rice plants for 2 days, and then kept on healthy rice seedling until check. The effects of dsRNA on expression of *NS4* and nucleoprotein *NSvc3* genes of RSV were performed by RT-PCR, as described previously [11], [13]. Furthermore, the accumulation of NS4 and RNPs of RSV in the bodies of SBPHs receiving dsRNAs were analysed by SDS-PAGE and immunoblotting with NS4- and RNP-specific antibodies, respectively, as reported previously [10], [28]. SBPH actins were detected with actin-specific antibodies. In additional, the internal organs from SBPHs receiving dsRNAs were fixed, labelled with RNP-rhodamine, NS4-FITC and actin dye phalloidin-Alexa Fluor 647 carboxylic acid (Invitrogen), and then examined by confocal microscopy, as described above.

To determine whether the treatment of dsRNAs would affect the transmission of RSV by the vector insects, SBPHs receiving dsRNAs were fed on diseased rice plants for 2 days, and then kept on healthy rice seedling for 15 days. For transmission assay, the individual SBPH was fed on healthy rice seedlings for 2 days. The viruliferous insects were checked by RT-PCR, as described above. The rice leaves were observed for visible symptoms.

### Examination of the effect of dsRNAs on viral replication in continuous cell cultures derived from SBPH

The continuous cell cultures derived from SBPH were established and maintained in the growth medium, as described previously [12]. Synchronous infection of SBPH vector cell in monolayer (VCM) by RSV was initiated as described previously [12]. To investigate the effect of synthesized dsRNAs on viral replication, VCMs were transfected with 0.5 µg/μl dsRNAs via cellfectin reagent (Invitrogen) for 24 h, and then were inoculated with RSV, as described previously [12]. VCMs were fixed with 4% paraformaldehyde for 30 min, labelled with NS4-FITC and RNP-rhodamine, and then examined by confocal microscopy, as described above. Furthermore, the accumulation of NS4 and RNPs of RSV in VCMs receiving dsRNAs were analysed by Western blot with NS4- and RNP-specific antibodies, respectively, as described above.

## Results

### Infection route of RSV in its vector insect as revealed by confocal microscopy

**Figure 1:**
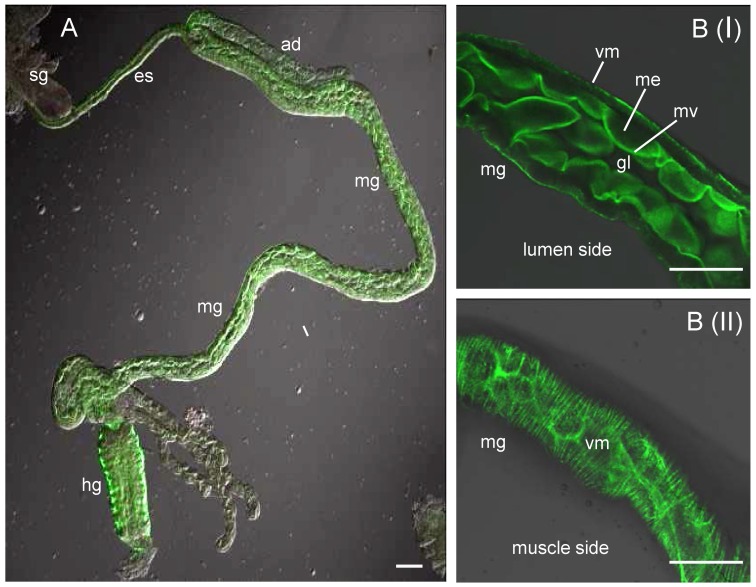
The digestive system SBPH. (A) The digestive system of SBPH consists of the salivary gland (sg) and the alimentary canal including the esophagus (es), anterior diverticulum (ad), midgut (mg) and hindgut (hg). (B) The single optical section of the lumen side (panel I) and muscle side (panel II) of the midgut. The internal organs of SBPHs were labelled for actin with phalloidin-FITC (green) and examined by confocal microscopy. gl, gut lumen; Mv microvilli; me, midgut epithelium; vm, visceral muscle. Bars, 100 µm.

**Figure 2:**
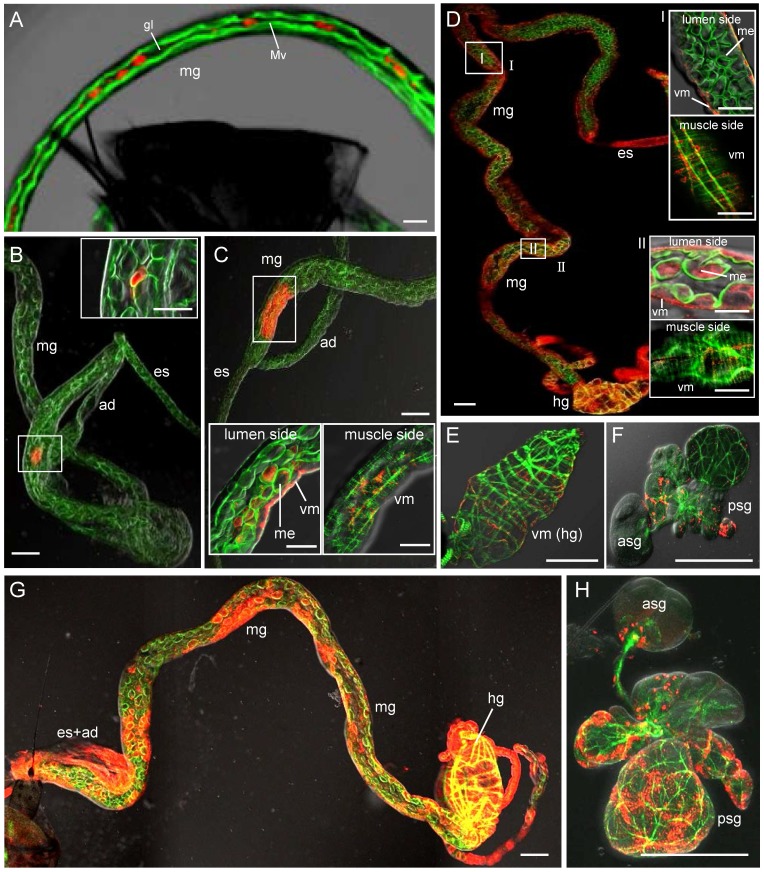
Infection route of RSV in the internal organs of its SBPH vector after ingestion of virus. Internal organs were labelled with RSV dye RNP-rhodamine (red) and actin dye phalloidin-FITC (green), and then examined with confocal microscopy. (A) At 1 day padp, RNP antigens were detected in the midgut lumen. (B) At 3 days padp, RNP antigens were detected in a single epithelial cell of the midgut. Inset: enlargement of the boxed area. (C) At 5 days padp, RNP antigens were detected in a limited number of epithelial cells and visceral muscle tissues of the midgut. Image was projected from 10 optical sections at 0.5 µm intervals. Insets: single optical section of different sides of the midgut. (D) At 8 days padp, RNP antigens were detected in the whole alimentary canal. Image was projected from 10 optical sections at 0.5 µm intervals. Insets I and II are enlarged images of the boxed areas I and II, respectively. Upper and lower panels of insets are each a single optical section of different sides of the midgut. (E) RNP antigens were detected in the visceral muscle tissues of the hindgut at 8 days padp. (F) RNP antigens were detected in the principal salivary glands, but not in the accessory salivary gland at 8 days padp. (G) At 14 days padp, RNP antigens were dense throughout the alimentary canal. (H) RNP antigens were detected in the salivary glands at 14 days padp. Images were merged with green fluorescence (actin) and red fluorescence (RNP antigens) under background visualized by transmitted light. es, esophagus; ad, anterior diverticulum; mg, midgut; hg, hindgut; asg, accessory salivary gland; psg, principal salivary glands; gl, gut lumen; Mv microvilli; me, midgut epithelium; vm, visceral muscle. Bars in panels A-F and H, 100 µm. Bars in panels G and I, and insets in panels B-D, 50 µm.

**Figure 3:**
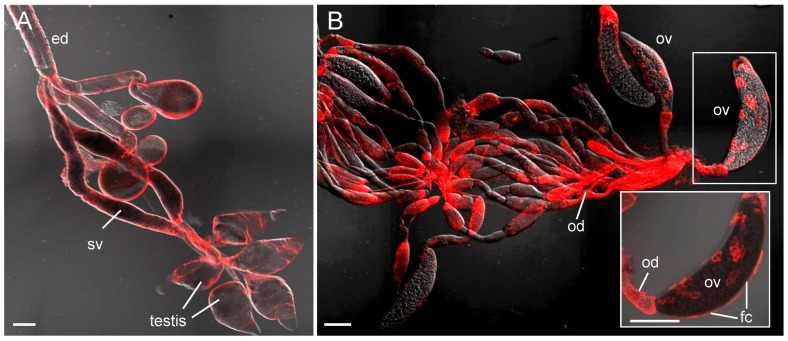
Accumulation of RNPs of RSV in reproductive system of viruliferous SBPHs. At 14 days padp, reproductive organs were labelled with RSV dye RNP-rhodamine and then examined with confocal microscopy. RNP antigens were detected in the testis, seminal vesicles and ejaculatory duct of males (panel A) and in the ovarioles and oviduct of females (panel B). Inset in panel B: enlargement of boxed area to show RNPs of RSV present in the follicular cells of ovariole. Images are shown with red fluorescence (RNP antigens) under background visualized by transmitted lights. ed, ejaculatory duct; sv, seminal vesicles; fc, follicular cells of ovariole; ov, ovariole; od, oviduct. Bars, 100 µm.

The infection route of RSV in its vector insect was examined by immunofluorescence microscopy. About 30% of SBPHs become viruliferous after a latent period of about 14 days (data not shown). Here, at 1, 3, 5, 8 and 14 days post-first access to diseased plants (padp), the internal organs from 50 SBPHs were examined with immunofluorescence microscopy. RSV was labelled with RNP-rhodamine; actin was labelled with actin dye phalloidin-FITC to show the morphology of the internal organs of SBPHs. As described previously [21], [30], the alimentary canal of SBPHs consists of the esophagus, anterior diverticulum, midgut and hindgut (Fig. 1A).The midgut epithelium of SBPHs was surrounded by visceral muscle tissues (Fig. 1B). At 1 day padp, RSV was accumulated in the midgut lumen in about 56% of SBPHs (Table 1, Fig. 2A). Three days padp, RSV was firstly observed in single midgut epithelial cell in about 14% of SBPHs (Table 1, Fig. 2B), suggesting that the midgut epithelium was the initial infection site for RSV. Five days padp, RSV had spread from the initial infection site, forming the small infection foci including 10–12 midgut epithelial cells in about 20% of SBPHs (Table 1, Fig. 2C). At this time, RSV had spread to the visceral muscle tissues surrounding the infected midgut epithelial cells (Fig. 2C). Eight days padp, RSV had spread to the entire alimentary canal in about 28% of SBPHs (Table 1, Fig. 2D, E). At this time, RSV had spread into the principal salivary glands in about 6% of SBPHs (Table 1, Fig. 2F). Fourteen days padp, RSV had extensively spread to the entire digestive system including the alimentary canal and salivary glands in about 24% of SBPHs (Table 1, Fig. 2G, H). At this time, in about 6% of female or 4% of male SBPHs, RSV was observed in the reproductive system: in the ovarioles and oviduct of females or in the testis, seminal vesicles and ejaculatory duct of males (Table 1, Fig. 3). Taken together, the sequential infection studies indicated that RSV initially infected the epithelial cells of the midgut, moved to the visceral muscles bordering the infected epithelium regions, then spread to the entire alimentary canal, and finally into the salivary glands and reproductive system.

**Table 1:** The distribution of RNP antigens of RSV in different tissues of SBPHs as revealed by immunofluorescence microscopy.

Tissues examined	No. of positive insects with RNPs of RSV in different tissues at different days padp (*n* = 50)				
	1 day	3 days	5 days	8 days	14 days
Midgut lumen	28	0	0	0	0
Midgut epithelium	0	7	10	14	4
Visceral muscle (midgut)	0	0	4	14	14
Visceral muscle (hindgut)	0	0	0	9	14
Esophagus	0	0	0	10	14
Anterior diverticulum	0	0	0	7	14
Salivary gland	0	0	0	3	14
Testes (males)	0	0	0	0	2
Ovarioles (females)	0	0	0	0	3

### Association of RNPs of RSV with NS4-specific inclusions in the vector insect

**Figure 4:**
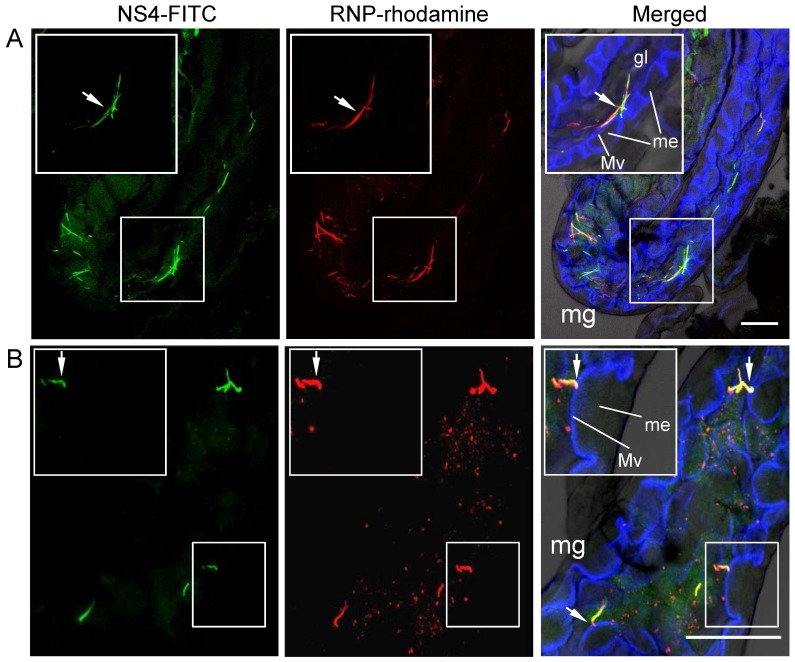
Association of RNPs of RSV with fibrillar inclusions of NS4 in midgut lumen of SBPH after ingestion of virus from diseased rice plants. At 1 day padp, the alimentary canal of SBPH was labelled with NS4-FITC (green), RNP-rhodamine (red) and actin dye Phalloidin-Alexa Fluor 647 carboxylic acid (blue), and then examined with confocal microscopy. (A) Colocalization of RNPs of RSV with fibrillar inclusions of NS4 in midgut lumen. (B) Fibrillar inclusions containing complex of RNPs and NS4, attached to actin-based microvilli of epithelial cells of midgut. Arrows indicate the attachment site of fibrillar inclusion on microvilli. Insets: enlarged images of boxed areas. Images were merged with green fluorescence (NS4 antigens), red fluorescence (RNP antigens) and blue fluorescence (actin dye) under background visualized by transmitted light. mg, midgut; gl, gut lumen; Mv microvilli; me, midgut epithelium. Bars, 100 µm.

**Figure 5:**
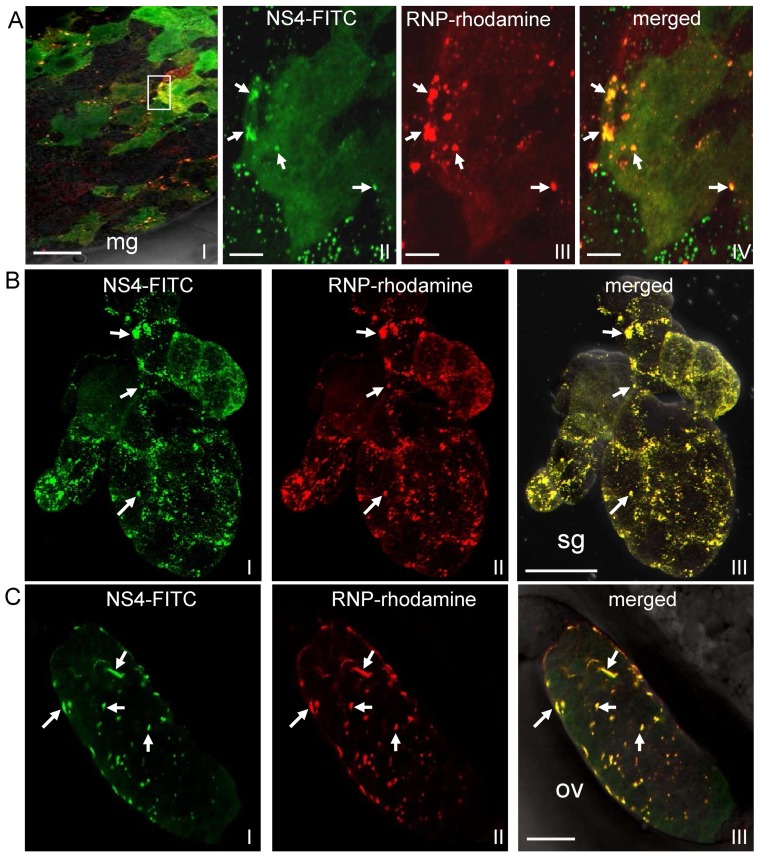
Association of RNPs of RSV with NS4-specific inclusions in the body of SBPH. Internal organs were labelled with NS4-FITC (green), RNP-rhodamine (red) and then examined with confocal microscopy. RNPs of RSV (red) colocalized with NS4 inclusions (green) in the midgut epithelium at 6 days padp (panel A), in the salivary glands at 12 days padp (panel B), and in the ovarioles of female at 12 days padp (panel C). Arrows: sites of colocalization. Frames II-IV in panel A are enlargements of boxed area in frame I. Frames I and IV in panel A, frames III in panels B and C are images merged with green fluorescence (NS4 antigens) and red fluorescence (RNP antigens) under background visualized by transmitted light. mg, midgut; sg, salivary gland; ov, ovariole. Bar in frame I of panel A, 10 µm. Bars in frames II-IV of panel A, 2 µm. Bars in panels B, C, 50 µm.

To determine whether the nonstructural protein NS4 can support viral infection in the body of viruliferous SBPH vectors, we investigated the distribution of NS4-specific inclusions and RNPs of RSV by double-labeling of the organs with NS4-FITC and RNP-rhodamine. By 1 day padp, NS4 formed fibrillar inclusions in the midgut lumen (Fig. 4A); RNPs of RSV were observed in punctate or fibrillar inclusions in the midgut lumen (Fig. 4A). The fibrillar inclusions of RNPs were seen to colocalize with the fibrillar inclusions of NS4 (Fig. 4A). The complex of RNPs and NS4 in the lumen was apparently able to attach to the actin-based microvilli of the midgut epithelium (Fig. 4B). It is clear that these inclusions had been ingested from the diseased rice plants. During viral infection in the body of viruliferous SBPHs, by 6 or 12 days padp, RNPs of RSV were observed to be closely associated with NS4-specific inclusions in the midgut epithelium, salivary glands and ovarioles of females (Fig. 5). Taken together, our results suggested that NS4-specific inclusions might support viral entry or infection in the body of viruliferous SBPHs.

**Figure 6:**
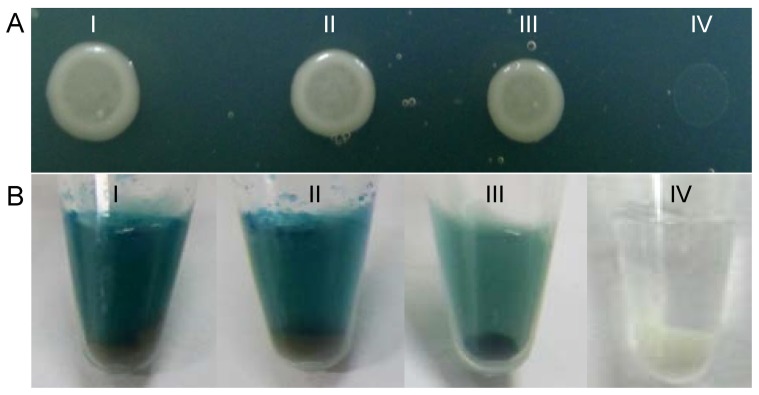
The interaction between NS4 and nucleoprotein NSvc3 of RSV detected by yeast two-hybrid assay. (A) Transformants on SD-trp-leu-his-ade agar plate. (B) The color in the HTX b-galactosidase assay. Frames I, Positive transformant of pTSU2-APP/pNubG-Fe65. Frames II, Transformant of pBT3-STE-NS4/pPR3-N-NSvc3. Frames III, Transformant of pBT3-STE-NSvc3/pPR3-N-NS4. Frames IV, Negative transformant of pTSU2-APP/pPR3-N.

The close association of NS4-specific inclusions with RNPs of RSV in the body of viruliferous SBPH vector suggested that NS4 might directly interact with nucleoprotein NSvc3 of RSV. To test this hypothesis, we used the yeast two-hybrid approach to detect the interaction between NS4 and nucleoprotein NSvc3 of RSV. The results showed that the yeasts expressing the combination of STE-NS4 and N-NSvc3 or the combination of STE-NSvc3 and N-NS4 were grown on SD-trp-leu-his-ade agar plates (Fig. 6). All these results confirmed that the formation of the complex of NS4 and RNPs of RSV in the body of viruliferous SBPH through direct NS4-NSvc3 interaction.

### RNAi induced by dsRNA from the *NS4* gene caused slower viral spread in the bodies of SBPHs

**Figure 7:**
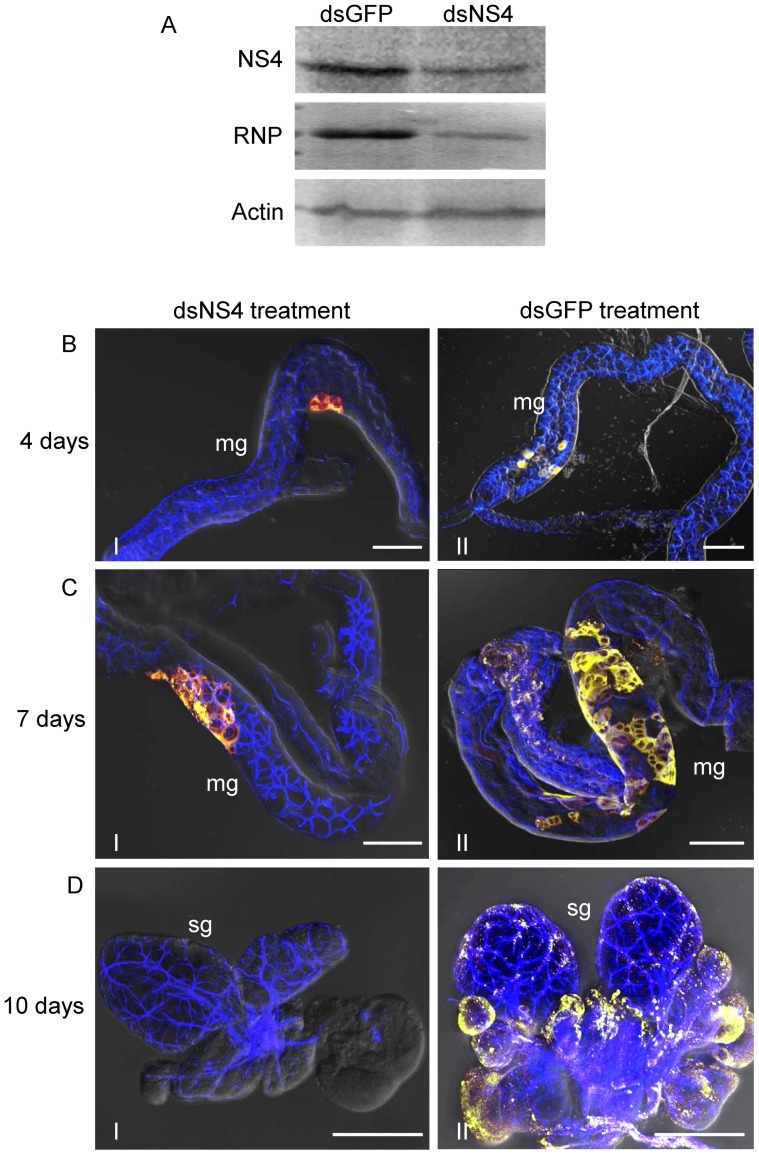
Ingestion of dsNS4 via membrane feeding by SBPHs suppressed the spread of RSV *in vivo*. (A) The treatment of dsNS4 significantly reduced the expression of RSV NS4 and the accumulation of RNPs of RSV in SBPHs, as revealed by Western blots assay. Protein extracts from SBPHs receiving dsRNAs were separated by SDS-PAGE to detect RNPs or NS4 with RNP- or NS4-specific antibodies, respectively. Insect actin was detected with actin-specific antibodies as a control. (B-D) Second instar nymphs of SBPHs were fed with dsNS4 (frames I) or dsGFP (frames II). At 4 (panel B), 7 (panel C) and 10 (panel D) days padp, internal organs of SBPHs were labelled with NS4-FITC (green), RNP-rhodamine (red), and actin dye phalloidin-Alexa Fluor 647 carboxylic acid (blue). Images with green fluorescence (NS4 antigens), red fluorescence (RNP antigens) and blue fluorescence (actin dye) were merged under background of transmitted light. mg, midgut; sg, salivary gland. Bars in panels B, C, 100 µm. Bars in panel D, 50 µm.

To further investigate the functional role of NS4 of RSV in the process of the transmission of RSV by SBPHs, second-instar nymphs of SBPHs were fed with dsRNAs from GFP gene (dsGFP) and dsRNAs from *NS4* gene (dsNS4) via membrane feeding for 1 day, allowed a 2-day acquisition on RSV-infected rice plants, and then kept on rice seedlings. At 10 days padp, RT-PCR test showed that about 28% of SBPHs receiving dsGFP were positive for the transcripts of *NS4* and *NSvc3* genes of RSV (Table 2). In contrast, only about 11% of SBPHs receiving dsNS4 were positive for the transcripts of *NS4* and *NSvc3* genes of RSV (Table 2). The effects of dsNS4 on the accumulation of NS4 and RNPs of RSV were further analysed by immunoblotting with NS4- and RNP-specific antibodies, respectively. In agreement with the RT-PCR results, the treatment with dsNS4 resulted in a significant reduction of the accumulation of NS4 and RNPs of RSV in the bodies of SBPHs (Fig. 7A). Thus, RNAi induced by dsNS4 could efficiently inhibit the accumulation of RSV in the bodies of SBPHs.

**Table 2:** Ingestion of dsNS4 via membrane feeding by SBPHs suppressed the accmulation of RSV *in vivo* and subsequent transmission of RSV by SBPHs.

Insects	No. of positive insects with *NS4* and *NSvc3* genes detected by RT-PCR at 10 days padp (*n* = 100)			No. of positive insects with RNPs and NS4 antigens detected by immunofluorescence microscopy at 4, 7 or 10 days padp (*n* = 50)				No. of positive insects that transmitted RSV at 15 days padp (*n* = 100)		
	Exp. no.			padp (days)	Limited area of midgut	Extensive area of midgut	salivary gland	Exp. no.		
	I	II	III					I	II	III
dsGFP	26	29	30	4	8	0	0	17	14	16
				7	5	7	0			
				10	2	12	8			
dsNS4	9	13	11	4	7	0	0	6	4	4
				7	10	2	0			
				10	6	5	4			

To further determine how the treatment with dsNS4 could inhibit the accumulation of RSV in the bodies of SBPHs, at 4, 8 and 12 days padp, internal organs from 50 SBPHs receiving dsRNAs were examined by immunofluorescence with the double-labeling of NS4-FITC and RNP-rhodamine. Four days padp, viral infection was restricted to a limited number of the midgut epithelial cells in 16% of SBPHs receiving dsGFP or dsNS4 (Table 2, Fig. 7B), suggesting that the treatment of dsNS4 did not inhibit the early infection of RSV in the midgut epithelium. Seven days padp, among SBPHs receiving dsGFP, 8% had infections in a limited area of midgut, and 16% in an extensive area of midgut. However, among SBPHs receiving dsNS4, 20% had infections in a limited area of midgut, and 4% in an extensive area of midgut (Table 2, Fig. 7C). Ten days padp, infections were observed in the salivary glands in 16% of SBPHs receiving dsGFP, but only in 4% of SBPHs receiving dsNS4 (Table 2, Fig. 7D). Therefore, our results suggested that the treatment of dsNS4 efficiently inhibited the spread of RSV from the early infection sites in the midgut epithelial cells of SBPHs, thus significantly preventing the spread of RSV into the salivary glands, from which RSV can be transmitted to rice plants. As expected, the transmission test showed that the treatment of dsNS4 efficiently inhibited the transmission of RSV by the vector insects (Table 2).

### RNAi induced by dsRNA from the *NS4* gene did not significantly affect viral replication in continuous cell culture derived from SBPH

**Figure 8:**
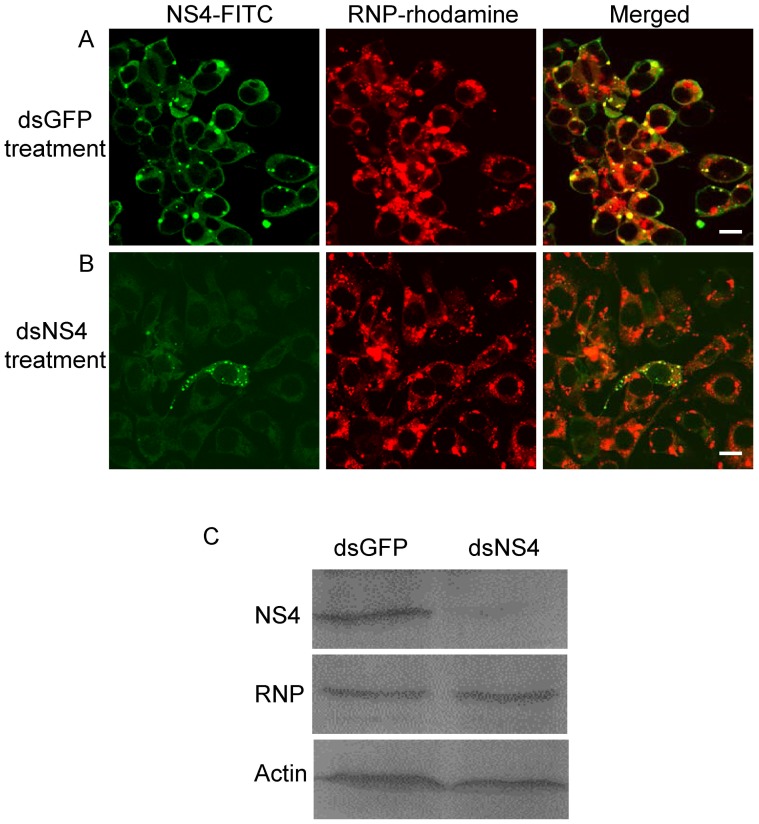
RNAi induced by dsNS4 suppressed the formation of NS4-specific inclusions without significant effect on RSV replication in VCMs. Twenty-four hours after transfection with dsGFP (A) or dsNS4 (B), VCMs were inoculated with RSV. Five days after viral inoculation, VCMs were labeled with NS4-FITC (green) and RNP-rhodamine (red). Images are representative of multiple experiments with multiple preparations. Bars, 10 µm. (C) Western blot analysis showed that the expression of NS4 but not RNPs of RSV was inhibited by RNAi induced by dsNS4 in virus-infected VCMs. Protein extracts from VCMs receiving dsRNAs were separated by SDS-PAGE to detect RNPs or NS4 with RNP- or NS4-specific antibodies, respectively. Insect actin was detected with actin-specific antibodies as a control.

To examine whether RNAi induced by dsNS4 affected viral replication, VCMs were transfected with dsRNAs. At 24 h after transfection, VCMs were inoculated with RSV. In VCMs transfected with dsGFP, viral infection was observed in almost 100% of cells, and NS4-specific inclusions were closely associated with RNPs of RSV (Fig. 8A). In VCMs transfected with dsNS4, viral infection still was observed in almost 100% of cells and the accumulation of RNPs of RSV was not significantly reduced, but the formation of NS4-specific inclusions was significantly inhibited (Fig. 8B). Furthermore, Western blot analysis showed that the transfection with dsNS4 resulted in a significant reduction of NS4 expression, but did not clearly affected the accumulation of RNPs of RSV in virus-infected VCMs (Fig. 8C). Taken together, all these results suggested that the inhibition of NS4-specific inclusions formation did not significantly affect viral replication in virus-infected insect vector cells.

## Discussion

Previously, the distribution of RNP antigens of RSV in the viruliferous SBPH had been investigated by immunoelectron microscopy [31], [32]. RNPs of RSV were localized in the cytoplasm in several tissues of the vector insect, including the midgut epithelium, salivary glands and follicular cells of the ovarioles [31], [32]. In the present study, the infection route of RSV in the bodies of SBPHs was investigated using immunofluoresence microscopy. Our observations showed that only a single epithelial cell of the midgut was susceptible to infection by RSV as early as 3 days padp, suggesting that there were only a limited number of cellular receptors on the microvillar membrane of the midgut for the attachment of RSV. In the early infection site, RSV would replicate and assemble abundant progeny RNPs, which spread to adjacent epithelial cells or progressed to the visceral muscle tissues, and finally spread into the salivary glands or reproductive system (Table 1, Fig. 2). Our recent findings revealed a similar infection route for RRSV, an orzavirus, in the body of its brown planthopper vector [21]. Both viruses rapidly crossed the initially infected epithelial cells of midgut into the visceral muscle tissues, from where viruses either spread throughout the visceral muscles of the midgut and hindgut, or directly disseminated into the hemolymph and finally into the salivary glands.

The formation of viral inclusion is essential for the replication or spread of persistent-propagative viruses in the bodies of vector insects [1], [2]. The replication of RSV induced the formation of FEO or ASO inclusion bodies composed of NS4 in host plants and vector insects [16], [17]. The fluorescent fibrillar inclusions of NS4 (Fig. 4), corresponding to FEO inclusion bodies in electron micrographs [16], were ingested by vector insects from diseased plants and accumulated in the lumen of midgut (Fig. 4). The association of RNPs of RSV with NS4-specific inclusion bodies in the lumen or on the microvillar membrane of the midgut came as a surprise (Fig. 4), suggesting that NS4 might aid RNPs in penetrating the epithelial cells of the midgut to initiate viral replication. During viral infection of the SBPH, RNPs of RSV were also closely associated with fluorescent punctate inclusions of NS4 (Fig. 5), which correspond to ASO inclusion bodies in electron micrographs [16], confirming previous hypothesis that NS4-specific ASO inclusion bodies might be involved in viral replication or spread in the body of SBPH. Furthermore, NS4 of RSV had been shown to specifically bind with purified RNPs in *in vitro* binding experiments [29]. Our observations confirmed that NS4 of RSV specifically interact with nucleoprotein NSvc3 of RSV in yeast two-hybrid assay (Fig. 6). All these results provided biochemical evidence for the formation of a complex between NS4 and RNPs through direct interaction with each other in the bodies of viruliferous SBPHs.

To gain direct evidence that NS4-specific inclusions play a critical role in the replication or spread of RSV in the body of SBPH, RNAi technique was used to interrupt the formation of NS4-specifc inclusions, then analyzed its effect on viral accumulation in the intact insect vectors. The insect was previously shown to autonomously take up dsRNA through feeding and digestion into the epithelia cells of its midgut, where RNAi machinery induced by dsRNA knocked down the expression of mRNA in a sequence-specific way [33], [34]. Thus, it is impossible that RNAi induced by dsNS4 could degrade the inclusions of NS4 ingested from diseased plants and block the entry of RNPs into epithelial cells from lumen. RNAi induced by ingestion of dsNS4 did not interfere with the early accumulation of RSV in the epithelial cells of the midgut (Table 2, Fig. 7B), suggesting that RNAi induced by dsNS4 in the epithelial cells of the midgut of SBPHs might not affect viral replication in the vector insects. The use of continuous cell culture of vector insect, an *in vitro* cultured monolayer system, would enable a synchronous and total cell response to viral infection and the treatment of the synthesized dsRNAs [12], [20], [35]. In this study, the continuous cell culture of SBPH was used to investigate the effect of dsNS4 on the replication of RSV in its insect vector cells. Our results showed that RNAi induced by dsNS4 specifically inhibited the formation of NS4-specific inclusions without a significant affect on the efficient replication of RSV in continuous cell culture of SBPH (Fig. 8). However, RNAi induced by dsNS4 strongly inhibited the spread of RSV from the initially infected cells to adjacent epithelial cells of the midgut and other organs such as the salivary gland in viruliferous SBPHs (Table 2, Fig. 7). Because RNAi induced by synthesized dsRNA *in vitro* is a sequence-specific gene-silencing mechanism [20], [21], [28] and the NS4 gene has no sequence homology with other RSV genes (data not shown), we deduced that RNAi induced by dsNS4 could specifically inhibited the formation of NS4-specific inclusions in SBPHs. Based on the analysis above, we determined that the slower spread of RSV in the body of SBPHs that received dsNS4 was directly caused by a significant loss in the functional role of NS4-specific inclusions in viral spread from initially infected epithelial cells of the midgut. Taken together, all these results suggested that NS4 of RSV played a critical role in viral spread in the body of SBPHs. In addition, the evidence that the knockdown of NS4 expression significantly reduced the transmissibility of RSV by SBPHs (Table 2), demonstrated that NS4 of RSV would enable to accomplish a latent period for RSV in the body of SBPHs.
